# The genome sequence of Bottle Sedge, *Carex rostrata* Stokes

**DOI:** 10.12688/wellcomeopenres.23874.1

**Published:** 2025-03-17

**Authors:** Markus Ruhsam, Andy Griffiths, Royal Botanic Garden Edinburgh Genome Acquisition Lab, Darwin Tree of Life Barcoding collective, Plant Genome Sizing collective, Wellcome Sanger Institute Tree of Life Management, Samples and Laboratory team, Wellcome Sanger Institute Scientific Operations: Sequencing Operations, Wellcome Sanger Institute Tree of Life Core Informatics team, Tree of Life Core Informatics collective, Darwin Tree of Life Consortium

**Affiliations:** 1 Royal Botanic Garden Edinburgh, Edinburgh, Scotland, UK

**Keywords:** Carex rostrata, Bottle Sedge, genome sequence, chromosomal, Poales

## Abstract

We present a genome assembly from a specimen of *Carex rostrata* (Bottle Sedge; Streptophyta; Magnoliopsida; Poales; Cyperaceae). The genome sequence has a total length of 382.30 megabases. Most of the assembly is scaffolded into 35 chromosomal pseudomolecules. Four mitochondrial genome scaffolds were assembled, and the one plastid genome, with a length of 220.95 kilobases.

## Species taxonomy

Eukaryota; Viridiplantae; Streptophyta; Streptophytina; Embryophyta; Tracheophyta; Euphyllophyta; Spermatophyta; Magnoliopsida; Mesangiospermae; Liliopsida; Petrosaviidae; commelinids; Poales; Cyperaceae; Cyperoideae; Cariceae; *Carex*; *Carex* subgen. *Carex*; *Carex rostrata* Stokes (NCBI:txid241230)

## Background

**Figure 1.**
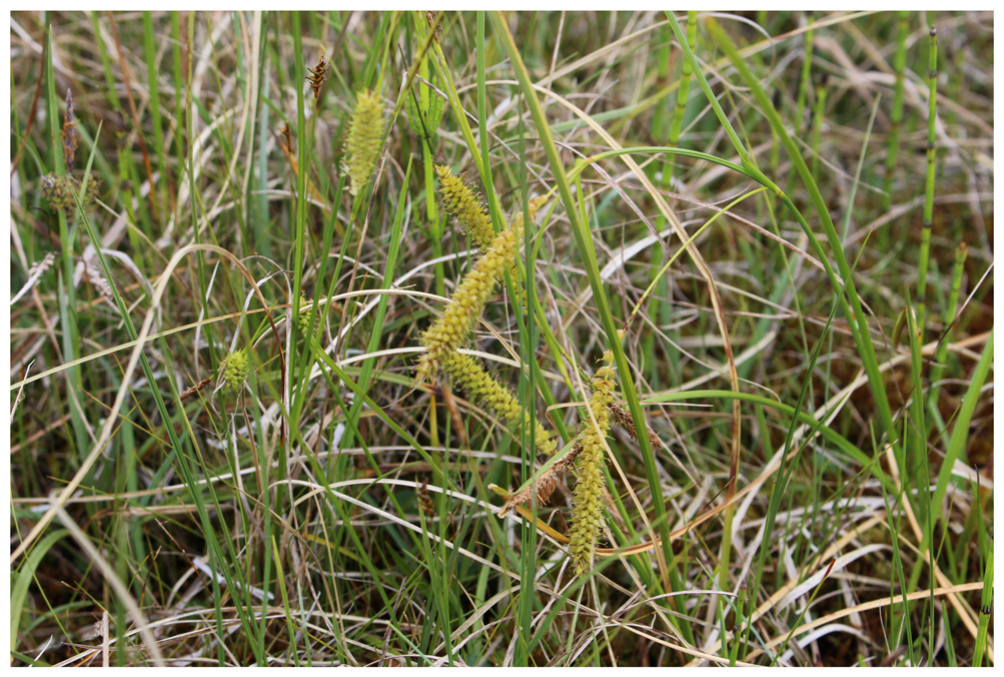
*Carex rostrata* population from which the sequenced specimen was taken.

The Bottle Sedge, *Carex rostrata* Stokes ( Figure 1), is a rhizomatous perennial sedge forming emergent stands on the margins of wet habitats such as lakes, rivers, fens, bogs, and flushes. Usually found around oligotrophic to mesotrophic acidic waters in can also occur in nutrient-poor calcareous conditions ( Stroh *et al.*, 2023). Bottle Sedge is widespread in Britain and Ireland and frequent in Scotland, Wales, northern England and Ireland. It has declined in south-eastern England and the Midlands due to wetland drainage and habitat destruction ( Stroh *et al.*, 2023). The species is part of the Circumpolar Boreo-temperate element with a distribution across the subarctic and temperate Eurasia and Subarctic America to northern United States of America ( POWO, 2024).

Here we present a chromosomally complete genome sequence for *Carex rostrata*, based on a specimen from Aberlady, United Kingdom, with chromosome number and genome size estimates that are consistent with previous reports ( Rotreklová *et al.*, 2011).

This genome for *Carex rostrata* will be a valuable resource for a breadth of future research. For example, this genome can increase our understanding of hybridisation within *Carex* and Cyperaceae. In Britain *C. rostrata* hybridises with *C. pseudocyprus* ( *C. x justi-schmidtii* Junge) and *C. vesicaria (C. x involute* (Bab.) Syme) and other hybrids are reported from the species broader distribution ( Jermy *et al.*, 2007). This reference genome could also facilitate investigation into mechanisms by which *C. rostrata* is able to tolerate elevated levels of metals such as zinc ( Matthews *et al.*, 2005) and water inundation ( Steed *et al.*, 2002).

The genome of the Bottle Sedge, *Carex rostrata*, was sequenced as part of the Darwin Tree of Life Project, a collaborative effort to sequence all named eukaryotic species in the Atlantic Archipelago of Britain and Ireland.

## Genome sequence report

### Sequencing data

The genome of a specimen of *Carex rostrata* was sequenced using Pacific Biosciences single-molecule HiFi long reads, generating 31.32 Gb (gigabases) from 2.12 million reads. GenomeScope analysis of the PacBio HiFi data estimated the haploid genome size at 403.48 Mb, with a heterozygosity of 1.35% and repeat content of 40.27%. These values provide an initial assessment of genome complexity and the challenges anticipated during assembly. Based on this estimated genome size, the sequencing data provided approximately 72.0x coverage of the genome. Hi-C sequencing produced 157.83 Gb from 1,045.25 million reads. Specimen and sequencing details are provided in Table 1.

**Table 1.** Specimen and sequencing data for *Carex rostrata*.

Project information			
**Study title**	*Carex rostrata*		
**Umbrella BioProject**	PRJEB68042		
**Species**	*Carex rostrata*		
**BioSpecimen**	SAMEA9335117		
**NCBI taxonomy ID**	241230		
Specimen information			
**Technology**	**ToLID**	**BioSample accession**	**Organism part**
**PacBio long read sequencing**	lpCarRost1	SAMEA9335201	Whole plant
**Hi-C sequencing**	lpCarRost1	SAMEA9335201	Whole plant
**RNA sequencing**	lpCarRost1	SAMEA9335201	Whole plant
Sequencing information			
**Platform**	**Run accession**	**Read count**	**Base count (Gb)**
**Hi-C Illumina NovaSeq 6000**	ERR12245634	1.05e+09	157.83
**PacBio Sequel IIe**	ERR12205301	2.02e+06	30.39
**PacBio Sequel IIe**	ERR12205300	7.24e+04	0.7
**PacBio Sequel IIe**	ERR12205302	2.01e+04	0.22
**RNA Illumina NovaSeq X**	ERR13093648	1.67e+08	25.24

### Assembly statistics

The primary haplotype was assembled, and contigs corresponding to an alternate haplotype were also deposited in INSDC databases. Assembly errors, including six missing joins or mis-joins and two haplotypic duplications, were corrected during manual curation. This increased the scaffold number by 4.9% and decreased the scaffold N50 by 2.9%. The final primary assembly has a total length of 382.30 Mb in 102 sequence scaffolds, with 46 gaps. The scaffold N50 is 12.5 Mb ( Table 2).

**Table 2.** Genome assembly data for *Carex rostrata*.

Genome assembly		
Assembly name	lpCarRost1.2	
Assembly accession	GCA_964058835.2	
*Alternate haplotype accession*	*GCA_964058875.1*	
Assembly level for primary assembly	chromosome	
Span (Mb)	381.00	
Number of contigs	119	
Number of scaffolds	73	
Longest scaffold (Mb)	20.75	
Assembly metric	Measure	*Benchmark*
Contig N50 length	6.59 Mb	*≥ 1 Mb*
Scaffold N50 length	12.53 Mb	*= chromosome N50*
Consensus quality (QV)	Primary: 62.9; alternate: 67.1; combined 64.5	*≥ 40*
*k*-mer completeness	Primary: 75.28%; alternate: 74.63%; combined: 98.60%	*≥ 95%*
BUSCO *	C:76.2%[S:69.0%,D:7.2%], F:2.1%,M:21.8%,n:4,896	*S > 90%*; *D < 5%*
Percentage of assembly mapped to chromosomes	98.7%	*≥ 90%*
Organelles	Mitochondrial genome: 1390.23 kb, Plastid genome: 220.95 kb	*complete single alleles*

* BUSCO scores based on the poales_odb10 BUSCO set using version 5.5.0. C = complete [S = single copy, D = duplicated], F = fragmented, M = missing, n = number of orthologues in comparison.

**Figure 2.**
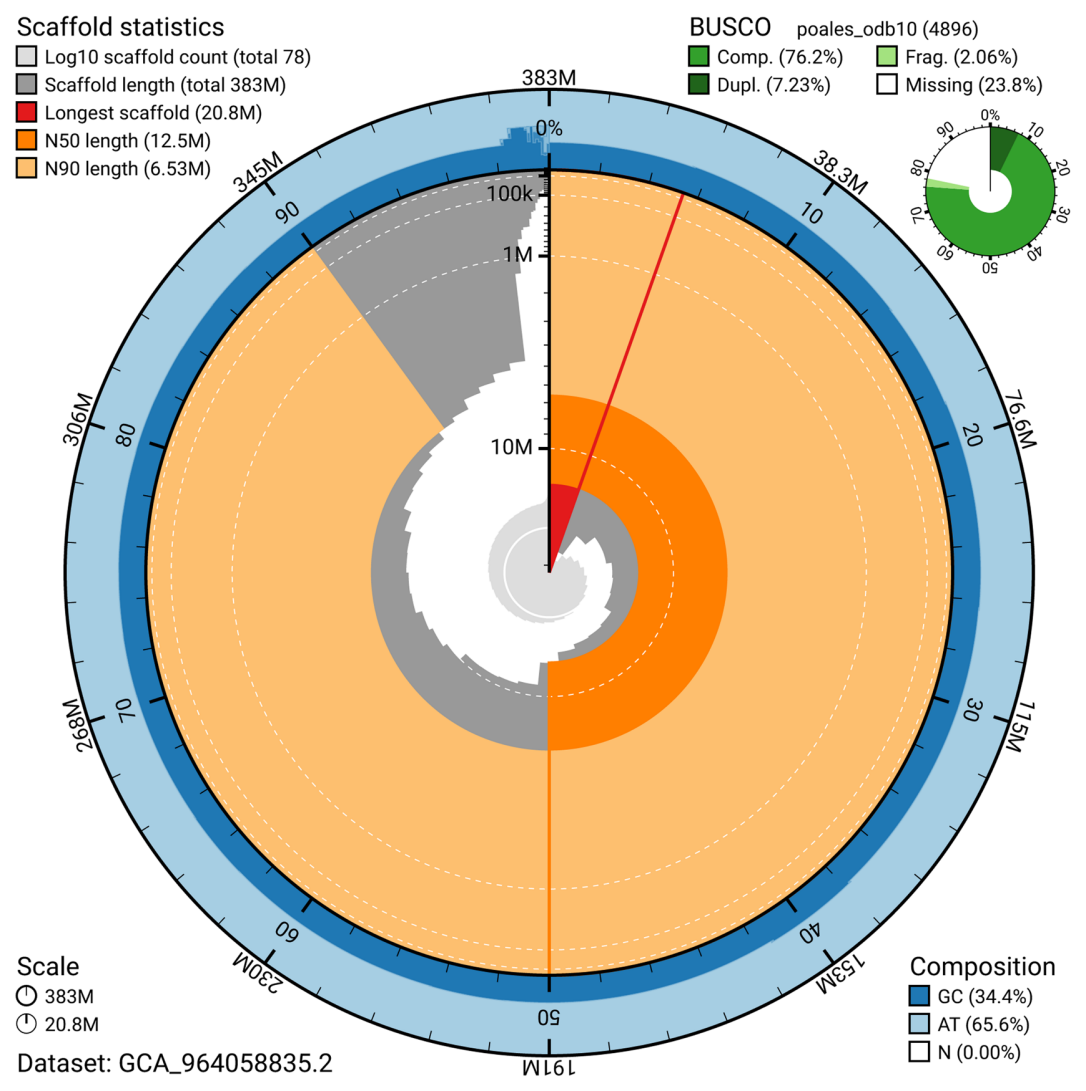
Genome assembly of *Carex rostrata*, lpCarRost1.2: metrics. The BlobToolKit snail plot provides an overview of assembly metrics and BUSCO gene completeness. The circumference represents the length of the whole genome sequence, and the main plot is divided into 1,000 bins around the circumference. The outermost blue tracks display the distribution of GC, AT, and N percentages across the bins. Scaffolds are arranged clockwise from longest to shortest and are depicted in dark grey. The longest scaffold is indicated by the red arc, and the deeper orange and pale orange arcs represent the N50 and N90 lengths. A light grey spiral at the centre shows the cumulative scaffold count on a logarithmic scale. A summary of complete, fragmented, duplicated, and missing BUSCO genes in the poales_odb10 set is presented at the top right. An interactive version of this figure is available at https://blobtoolkit.genomehubs.org/view/GCA_964058835.2/dataset/GCA_964058835.2/snail.

**Figure 3.**
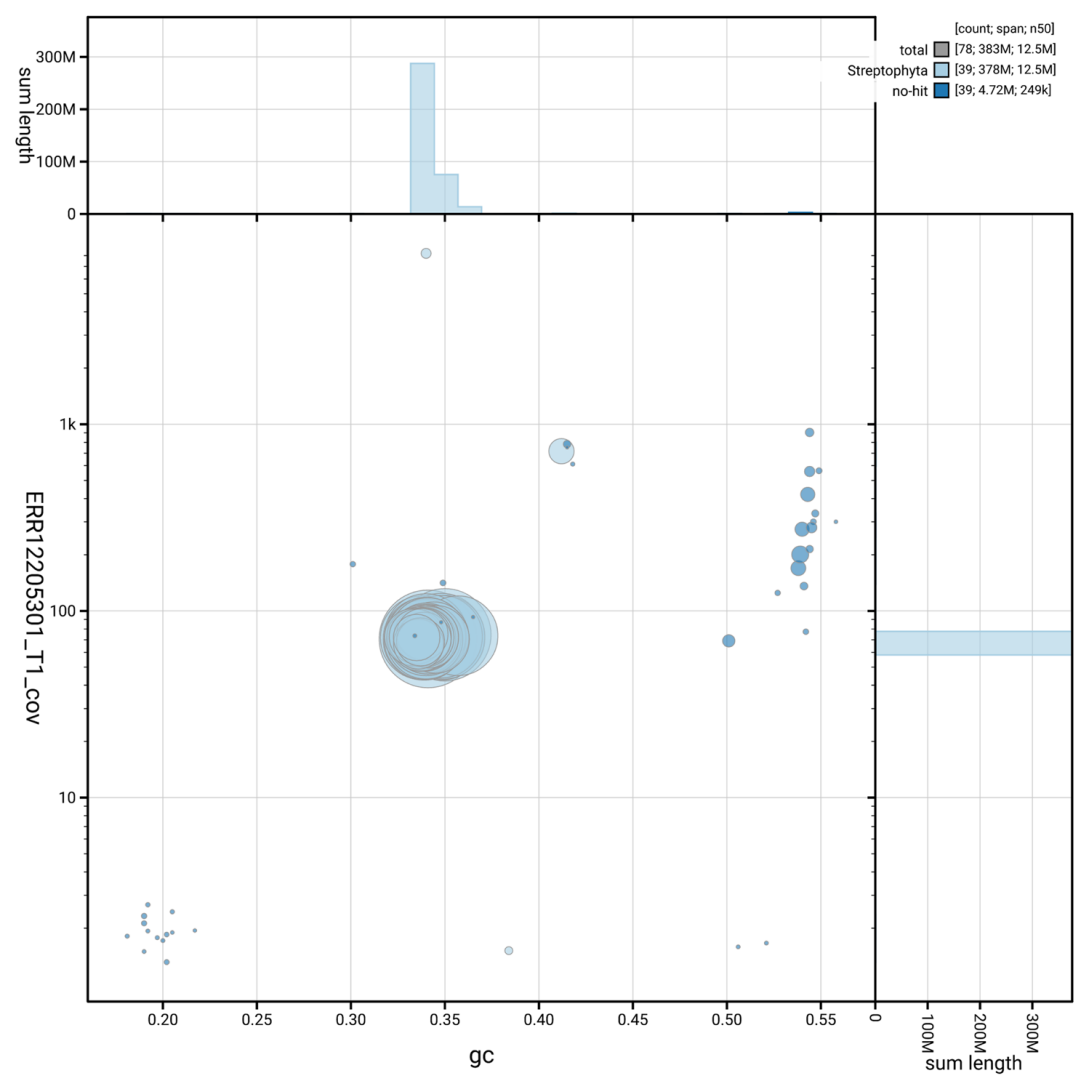
Genome assembly of *Carex rostrata*, lpCarRost1.2: BlobToolKit GC-coverage plot. Blob plot showing sequence coverage (vertical axis) and GC content (horizontal axis). The circles represent scaffolds, with the size proportional to scaffold length and the colour representing phylum membership. The histograms along the axes display the total length of sequences distributed across different levels of coverage and GC content. An interactive version of this figure is available at https://blobtoolkit.genomehubs.org/view/GCA_964058835.2/dataset/GCA_964058835.2/blob.

**Figure 4.**
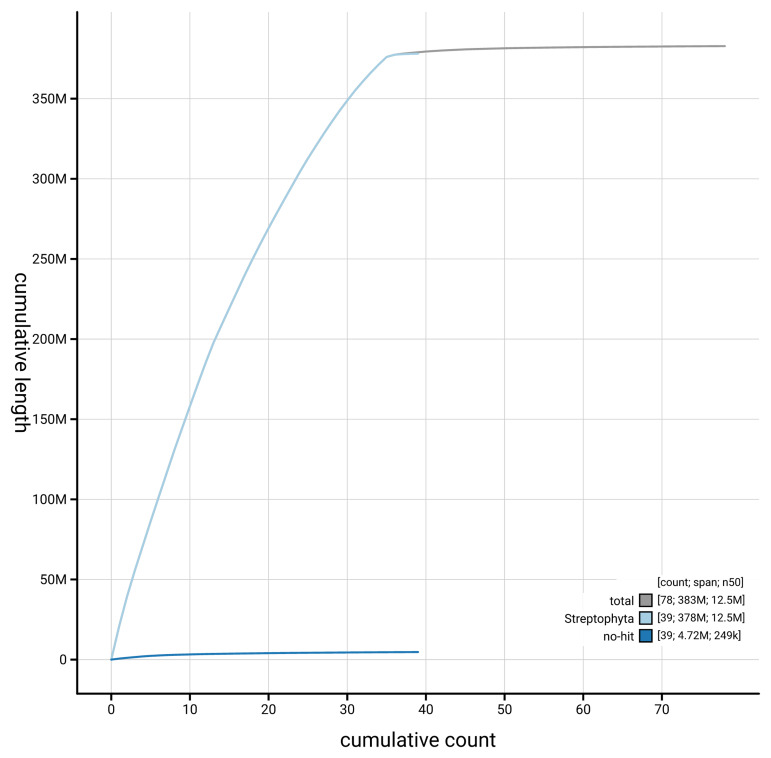
Genome assembly of *Carex rostrata,* lpCarRost1.2: BlobToolKit cumulative sequence plot. The grey line shows cumulative length for all scaffolds. Coloured lines show cumulative lengths of scaffolds assigned to each phylum using the buscogenes taxrule. An interactive version of this figure is available at https://blobtoolkit.genomehubs.org/view/GCA_964058835.2/dataset/GCA_964058835.2/cumulative.

**Figure 5.**
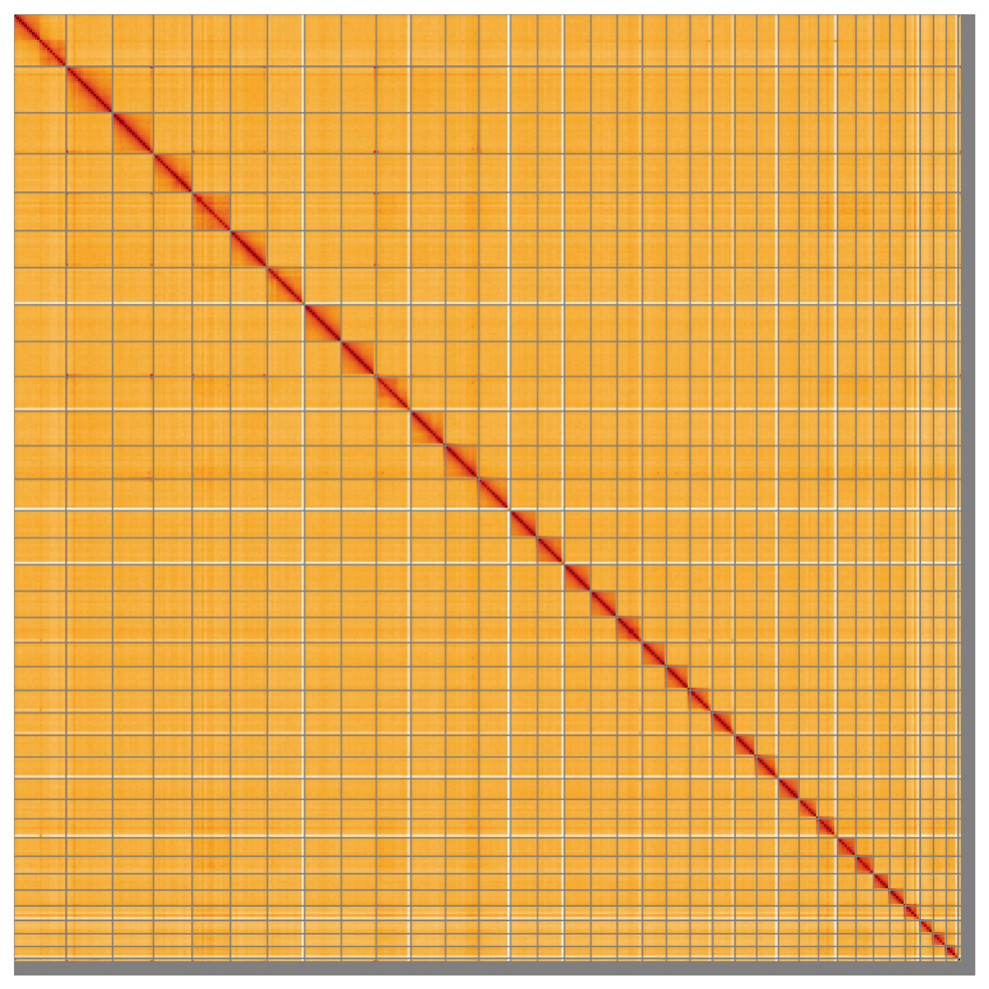
Genome assembly of *Carex rostrata*, lpCarRost1.2: Hi-C contact map of the lpCarRost1.2 assembly, visualised using HiGlass. Chromosomes are shown in order of size from left to right and top to bottom. Darker shades indicate more frequent physical contacts between regions, while lighter areas represent fewer contacts. An interactive version of this figure may be viewed at https://genome-note-higlass.tol.sanger.ac.uk/l/?d=VPxfwOKuSyedaNvzyXsUnw.

The snail plot in Figure 2 summarises the assembly statistics, while the blob plot in Figure 3 shows the distribution of assembly scaffolds by GC proportion and coverage. The cumulative assembly plot in Figure 4 shows curves for subsets of scaffolds assigned to different phyla. Most (97.89%) of the assembly sequence was assigned to 35 chromosomal-level scaffolds. Chromosome-scale scaffolds confirmed by the Hi-C data are named in order of size ( Figure 5; Table 3).

**Table 3.** Chromosomal pseudomolecules in the genome assembly of *Carex rostrata*, lpCarRost1.

INSDC accession	Name	Length (Mb)	GC%
OZ060077.1	1	20.75	34
OZ060078.1	2	18.54	35
OZ060079.1	3	16.35	35
OZ060080.1	4	15.29	34
OZ060081.1	5	15.28	35
OZ060082.1	6	14.81	35
OZ060083.1	7	14.73	34
OZ060084.1	8	14.64	34
OZ060085.1	9	13.94	34
OZ060086.1	10	13.82	35.5
OZ060087.1	11	13.72	34
OZ060088.1	12	13.36	34
OZ060089.1	13	12.53	34
OZ060090.1	14	10.82	34.5
OZ060091.1	15	10.63	34
OZ060092.1	16	10.56	34
OZ060093.1	17	10.55	34
OZ060094.1	18	9.96	34.5
OZ060095.1	19	9.64	34
OZ060096.1	20	9.39	34
OZ060097.1	21	8.96	34
OZ060098.1	22	8.87	33.5
OZ060099.1	23	8.72	34
OZ060100.1	24	8.68	33.5
OZ060101.1	25	8.22	34
OZ060102.1	26	7.69	34
OZ060103.1	27	7.6	33.5
OZ060104.1	28	7.29	34
OZ060105.1	29	7.01	33.5
OZ060106.1	30	6.53	33.5
OZ060107.1	31	6.29	34
OZ060108.1	32	5.73	34
OZ060109.1	33	5.43	33.5
OZ060110.1	34	5.03	33.5
OZ060111.1	35	4.69	33.5
OZ060116.1	Pltd	0.22	34
OZ060112.1	MT1	1.39	41
OZ060113.1	MT2	0.04	41.5
OZ060114.1	MT3	0.13	41.5
OZ060115.1	MT4	0.02	41.5

The mitochondrial and plastid genomes were also assembled and can be found as contigs within the multifasta file of the genome submission.

### Assembly quality metrics

The estimated Quality Value (QV) and *k*-mer completeness metrics, along with BUSCO completeness scores, were calculated for each haplotype and the combined assembly. The QV reflects the base-level accuracy of the assembly, while *k*-mer completeness indicates the proportion of expected *k*-mers identified in the assembly. BUSCO scores provide a measure of completeness based on benchmarking universal single-copy orthologues.

The primary haplotype has a QV of 62.9, and the combined primary and alternate assemblies achieve an estimated QV of 64.5. The *k*-mer recovery for the primary haplotype is 75.28%, and for the alternate haplotype 74.63%; the combined primary and alternate assemblies have a *k*-mer recovery of 98.60%. BUSCO v.5.5.0 analysis using the poales_odb10 reference set ( *n* = 4,896) identified 76.2% of the expected gene set (single = 69.0%, duplicated = 7.2%).

Table 2 provides assembly metric benchmarks adapted from Rhie *et al.* (2021) and the Earth BioGenome Project (EBP) Report on Assembly Standards September 2024. The primary assembly achieves the EBP reference standard of **6.C.Q63**.

## Methods

### Sample acquisition, DNA barcoding and genome size estimation

Stems and leaves were sampled from *Carex rostrata* (specimen ID EDTOL01368, ToLID lpCarRost1) collected from Aberlady, Scotland, United Kingdom (latitude 56.02, longitude –2.86) on 2021-06-14 (collection number DMR34). The specimen was collected and formally identified by Markus Ruhsam (Royal Botanic Garden Edinburgh) and preserved in liquid nitrogen. The herbarium voucher associated with the sequenced plant is https://data.rbge.org.uk/herb/E01152251 and is deposited in the herbarium of RBG Edinburgh (E).

The initial species identification was verified by an additional DNA barcoding process following the framework developed by Twyford *et al*. (2024). Part of the plant specimen was preserved in silica gel desiccant ( Chase & Hills, 1991). DNA was extracted from the dried specimen, then PCR was used to amplify standard barcode regions. The resulting amplicons were sequenced and compared to public sequence databases including GenBank and the Barcode of Life Database (BOLD). The barcode sequences for this specimen are available on BOLD ( Ratnasingham & Hebert, 2007). Following whole genome sequence generation, DNA barcodes were also used alongside the initial barcoding data for sample tracking through the genome production pipeline at the Wellcome Sanger Institute ( Twyford *et al.*, 2024). The standard operating procedures for the Darwin Tree of Life barcoding have been deposited on protocols.io ( Beasley *et al.*, 2023).

The genome size was estimated by flow cytometry using the fluorochrome propidium iodide and following the ‘one-step’ method outlined in Pellicer *et al.* (2021). For this species, CyStain™ PI OxProtect Staining Buffer (cat. No. 05-5027; Sysmex UK Ltd.) was used for isolation of nuclei ( Loureiro *et al.*, 2007), and the internal calibration standard was *Solanum lycopersicum* ‘Stupiké polní rané’ with an assumed 1C-value of 968 Mb ( Doležel *et al.*, 2007).

### Nucleic acid extraction

The workflow for high molecular weight (HMW) DNA extraction at the WSI Tree of Life Core Laboratory includes a sequence of core procedures: sample preparation and homogenisation, DNA extraction, fragmentation and purification. Detailed protocols are available on protocols.io ( Denton *et al.*, 2023). The lpCarRost1 sample was weighed and dissected on dry ice ( Jay *et al.*, 2023), and leaf and stem tissue was cryogenically disrupted using the Covaris cryoPREP ^®^ Automated Dry Pulverizer ( Narváez-Gómez *et al.*, 2023). HMW DNA was extracted using the Plant Organic Extraction protocol ( Jackson & Howard, 2023). HMW DNA was sheared into an average fragment size of 12–20 kb in a Megaruptor 3 system ( Bates *et al.*, 2023). Sheared DNA was purified by solid-phase reversible immobilisation, using AMPure PB beads to eliminate shorter fragments and concentrate the DNA ( Oatley *et al.*, 2023). The concentration of the sheared and purified DNA was assessed using a Nanodrop spectrophotometer and Qubit Fluorometer and Qubit dsDNA High Sensitivity Assay kit. Fragment size distribution was evaluated by running the sample on the FemtoPulse system.

RNA was extracted from tissue of lpCarRost1 in the Tree of Life Laboratory at the WSI using the RNA Extraction: Automated MagMax™ *mir*Vana protocol ( do Amaral *et al.*, 2023). The RNA concentration was assessed using a Nanodrop spectrophotometer and a Qubit Fluorometer using the Qubit RNA Broad-Range Assay kit. Analysis of the integrity of the RNA was done using the Agilent RNA 6000 Pico Kit and Eukaryotic Total RNA assay.

### Hi-C sample preparation and cross-linking

Hi-C data were generated from tissue of the lpCarRost1 sample at the WSI Scientific Operations core, using the Arima-HiC v2 kit. Tissue was finely ground using cryoPREP, and then subjected to nuclei isolation using a modified protocol of the Qiagen QProteome Kit. After isolation, the nuclei were fixed, and the DNA crosslinked using a 37% formaldehyde solution. The crosslinked DNA was then digested using the restriction enzyme master mix. The 5’-overhangs were then filled in and labelled with biotinylated nucleotides and proximally ligated. An overnight incubation was carried out for enzymes to digest remaining proteins and for crosslinks to reverse. A clean up was performed with SPRIselect beads prior to library preparation. DNA concentration was quantified using the Qubit Fluorometer v2.0 and Qubit HS Assay Kit according to the manufacturer’s instructions.

### Library preparation and sequencing

Library preparation and sequencing were performed at the WSI Scientific Operations core.

***PacBio HiFi***

At a minimum, samples were required to have an average fragment size exceeding 8 kb and a total mass over 400 ng to proceed to the low input SMRTbell Prep Kit 3.0 protocol (Pacific Biosciences, California, USA). Libraries were prepared using the SMRTbell Prep Kit 3.0 (Pacific Biosciences, California, USA) as per the manufacturer’s instructions. The kit includes the reagents required for end repair/A-tailing, adapter ligation, post-ligation SMRTbell bead cleanup, and nuclease treatment. Following the manufacturer’s instructions, size selection and clean up was carried out using diluted AMPure PB beads (Pacific Biosciences, California, USA). DNA concentration was quantified using the Qubit Fluorometer v4.0 (Thermo Fisher Scientific) with Qubit 1X dsDNA HS assay kit and the final library fragment size analysis was carried out using the Agilent Femto Pulse Automated Pulsed Field CE Instrument (Agilent Technologies) and gDNA 55kb BAC analysis kit.

Samples were sequenced on a Sequel IIe instrument (Pacific Biosciences, California, USA). The concentration of the library loaded onto the Sequel IIe was in the range 40–135 pM. The SMRT link software, a PacBio web-based end-to-end workflow manager, was used to set-up and monitor the run, as well as perform primary and secondary analysis of the data upon completion.

***Hi-C***

For Hi-C library preparation, DNA was fragmented to a size of 400 to 600 bp using a Covaris E220 sonicator. The DNA was then enriched, barcoded, and amplified using the NEBNext Ultra II DNA Library Prep Kit (New England Biolabs) following manufacturer’s instructions. Hi-C sequencing was performed using paired-end sequencing with a read length of 150 bp on an Illumina NovaSeq 6000 instrument.

***RNA***

Poly(A) RNA-Seq libraries were constructed using the NEB Ultra II RNA Library Prep kit, following the manufacturer’s instructions. RNA sequencing was performed on the Illumina NovaSeq X instrument.

### Genome assembly, curation and evaluation

***Assembly***

Prior to assembly of the PacBio HiFi reads, a database of *k*-mer counts ( *k* = 31) was generated from the filtered reads using FastK. GenomeScope2 ( Ranallo-Benavidez *et al.*, 2020) was used to analyse the *k*-mer frequency distributions, providing estimates of genome size, heterozygosity, and repeat content.

The HiFi reads were assembled using Hifiasm ( Cheng *et al.*, 2021) with the --primary option. Haplotypic duplications were identified and removed using purge_dups ( Guan *et al.*, 2020). The Hi-C reads were mapped to the primary contigs using bwa-mem2 ( Vasimuddin *et al.*, 2019). The contigs were further scaffolded using the provided Hi-C data ( Rao *et al.*, 2014) in YaHS ( Zhou *et al.*, 2023) using the --break option. The scaffolded assemblies were evaluated using Gfastats ( Formenti *et al.*, 2022), BUSCO ( Manni *et al.*, 2021) and MerquryFK ( Rhie *et al.*, 2020). The organelle genomes were assembled using OATK ( Zhou, 2023).

***Curation***

The assembly was decontaminated using the Assembly Screen for Cobionts and Contaminants (ASCC) pipeline (article in preparation). Flat files and maps used in curation were generated in TreeVal ( Pointon *et al.*, 2023). Manual curation was primarily conducted using PretextView ( Harry, 2022), with additional insights provided by JBrowse2 ( Diesh *et al.*, 2023) and HiGlass ( Kerpedjiev *et al.*, 2018). Scaffolds were visually inspected and corrected as described by Howe *et al*. (2021). Any identified contamination, missed joins, and mis-joins were corrected, and duplicate sequences were tagged and removed. The process is documented at https://gitlab.com/wtsi-grit/rapid-curation (article in preparation).

***Evaluation of assembly quality***

The Merqury.FK tool ( Rhie *et al.*, 2020), run in a Singularity container ( Kurtzer *et al.*, 2017), was used to evaluate *k*-mer completeness and assembly quality for the primary and alternate haplotypes using the *k*-mer databases ( *k* = 31) computed prior to genome assembly. The analysis outputs included assembly QV scores and completeness statistics.

A Hi-C contact map was produced for the final version of the assembly. The Hi-C reads were aligned using bwa-mem2 ( Vasimuddin *et al.*, 2019) and the alignment files were combined using SAMtools ( Danecek *et al.*, 2021). The Hi-C alignments were converted into a contact map using BEDTools ( Quinlan & Hall, 2010) and the Cooler tool suite ( Abdennur & Mirny, 2020). The contact map was visualised in HiGlass ( Kerpedjiev *et al.*, 2018).

The blobtoolkit pipeline is a Nextflow port of the previous Snakemake Blobtoolkit pipeline ( Challis *et al.*, 2020). It aligns the PacBio reads in SAMtools and minimap2 ( Li, 2018) and generates coverage tracks for regions of fixed size. In parallel, it queries the GoaT database ( Challis *et al.*, 2023) to identify all matching BUSCO lineages to run BUSCO ( Manni *et al.*, 2021). For the three domain-level BUSCO lineages, the pipeline aligns the BUSCO genes to the UniProt Reference Proteomes database ( Bateman *et al.*, 2023) with DIAMOND ( Buchfink *et al.*, 2021) blastp. The genome is also split into chunks according to the density of the BUSCO genes from the closest taxonomic lineage, and each chunk is aligned to the UniProt Reference Proteomes database with DIAMOND blastx. Genome sequences with no hits are chunked with seqtk and aligned to the NT database with blastn ( Altschul *et al.*, 1990). The blobtools suite combines all these outputs into a blobdir for visualisation.

The blobtoolkit pipeline was developed using nf-core tooling ( Ewels *et al.*, 2020) and MultiQC ( Ewels *et al.*, 2016), relying on the Conda package manager, the Bioconda initiative ( Grüning *et al.*, 2018), the Biocontainers infrastructure ( da Veiga Leprevost *et al.*, 2017), as well as the Docker ( Merkel, 2014) and Singularity ( Kurtzer *et al.*, 2017) containerisation solutions.

Table 4 contains a list of relevant software tool versions and sources.

**Table 4.** Software tools: versions and sources.

Software tool	Version	Source
BEDTools	2.30.0	https://github.com/arq5x/bedtools2
BLAST	2.14.0	ftp://ftp.ncbi.nlm.nih.gov/blast/executables/blast+/
BlobToolKit	4.3.9	https://github.com/blobtoolkit/blobtoolkit
BUSCO	5.5.0	https://gitlab.com/ezlab/busco
bwa-mem2	2.2.1	https://github.com/bwa-mem2/bwa-mem2
Cooler	0.8.11	https://github.com/open2c/cooler
DIAMOND	2.1.8	https://github.com/bbuchfink/diamond
fasta_windows	0.2.4	https://github.com/tolkit/fasta_windows
FastK	427104ea91c78c3b8b8b49f1a7d6bbeaa869ba1c	https://github.com/thegenemyers/FASTK
Gfastats	1.3.6	https://github.com/vgl-hub/gfastats
GoaT CLI	0.2.5	https://github.com/genomehubs/goat-cli
Hifiasm	0.19.5-r587	https://github.com/chhylp123/hifiasm
HiGlass	44086069ee7d4d3f6f3f0012569789ec138f42b84a a44357826c0b6753eb28de	https://github.com/higlass/higlass
MerquryFK	d00d98157618f4e8d1a9190026b19b471055b22e	https://github.com/thegenemyers/MERQURY.FK
Minimap2	2.24-r1122	https://github.com/lh3/minimap2
MultiQC	1.14, 1.17, and 1.18	https://github.com/MultiQC/MultiQC
Nextflow	23.10.0	https://github.com/nextflow-io/nextflow
OATK	1.0	https://github.com/c-zhou/oatk
PretextView	0.2.5	https://github.com/sanger-tol/PretextView
purge_dups	1.2.3	https://github.com/dfguan/purge_dups
samtools	1.19.2	https://github.com/samtools/samtools
sanger-tol/ascc	-	https://github.com/sanger-tol/ascc
sanger-tol/blobtoolkit	0.6.0	https://github.com/sanger-tol/blobtoolkit
Seqtk	1.3	https://github.com/lh3/seqtk
Singularity	3.9.0	https://github.com/sylabs/singularity
TreeVal	1.2.0	https://github.com/sanger-tol/treeval
YaHS	1.1a.2	https://github.com/c-zhou/yahs

### Wellcome Sanger Institute – Legal and Governance

The materials that have contributed to this genome note have been supplied by a Darwin Tree of Life Partner. The submission of materials by a Darwin Tree of Life Partner is subject to the **‘Darwin Tree of Life Project Sampling Code of Practice’**, which can be found in full on the Darwin Tree of Life website here. By agreeing with and signing up to the Sampling Code of Practice, the Darwin Tree of Life Partner agrees they will meet the legal and ethical requirements and standards set out within this document in respect of all samples acquired for, and supplied to, the Darwin Tree of Life Project.

Further, the Wellcome Sanger Institute employs a process whereby due diligence is carried out proportionate to the nature of the materials themselves, and the circumstances under which they have been/are to be collected and provided for use. The purpose of this is to address and mitigate any potential legal and/or ethical implications of receipt and use of the materials as part of the research project, and to ensure that in doing so we align with best practice wherever possible. The overarching areas of consideration are:

•  Ethical review of provenance and sourcing of the material

•  Legality of collection, transfer and use (national and international)

Each transfer of samples is further undertaken according to a Research Collaboration Agreement or Material Transfer Agreement entered into by the Darwin Tree of Life Partner, Genome Research Limited (operating as the Wellcome Sanger Institute), and in some circumstances other Darwin Tree of Life collaborators.

## Data Availability

European Nucleotide Archive: Carex rostrata. Accession number PRJEB68042; https://identifiers.org/ena.embl/PRJEB68042. The genome sequence is released openly for reuse. The *Carex rostrata* genome sequencing initiative is part of the Darwin Tree of Life (DToL) project. All raw sequence data and the assembly have been deposited in INSDC databases. The genome will be annotated using available RNA-Seq data and presented through the Ensembl pipeline at the European Bioinformatics Institute. Raw data and assembly accession identifiers are reported in Table 1.

## Author information

Members of the Royal Botanic Garden Edinburgh Genome Acquisition Lab are listed here: https://doi.org/10.5281/zenodo.4786682.

Members of the Plant Genome Sizing collective are listed here: https://doi.org/10.5281/zenodo.7994306.

Members of the Darwin Tree of Life Barcoding collective are listed here: https://doi.org/10.5281/zenodo.12158331

Members of the Wellcome Sanger Institute Tree of Life Management, Samples and Laboratory team are listed here: https://doi.org/10.5281/zenodo.12162482.

Members of Wellcome Sanger Institute Scientific Operations: Sequencing Operations are listed here: https://doi.org/10.5281/zenodo.12165051.

Members of the Wellcome Sanger Institute Tree of Life Core Informatics team are listed here: https://doi.org/10.5281/zenodo.12160324.

Members of the Tree of Life Core Informatics collective are listed here: https://doi.org/10.5281/zenodo.12205391.

Members of the Darwin Tree of Life Consortium are listed here: https://doi.org/10.5281/zenodo.4783558.
