# Supplementary material for: The genome sequence of Bottle Sedge, Carex rostrata Stokes
Source: Wellcome Open Res. 2025 Mar 17;10:132. [Version 1] doi: 10.12688/wellcomeopenres.23874.1 (PMC12635528; doi:10.12688/wellcomeopenres.23874.1)
Supplement: Peer review history

# Reviewer report: Reviewer response for version 1

**DOI:** 10.21956/wellcomeopenres.26340.r137676

**Published:** 2025-11-25

**Authors:** Marcial Escudero

**Affiliations:** 1 Universidad de Sevilla, Seville, Andalusia, Spain

This paper presents the genome assembly of a species from which there is no previous assembly. The assembly reaches the chromosome level with an adequate quality up to standard with other assemblies in this journal. The methods of both the dna extraction, hi-C protocol, sequencing and bioinformatic analysis are exposed with sufficient detail, ensuring that replication is possible. But some attention should be put into references in the methods. The quality analyses are shown clearly in a format suitable for this kind of journal, showing the good quality of the assembly. However, k value for kmer analysis has been set to 31 without explanation when the standard in 21. Some explanation of why 31 was set as the value of k, we believe it is need. Overall, we consider this paper is up to standard with other similar papers in this journal and can be therefore published.

 Here we include some comments and corrections that the authors may address before submitting:

 Firstly and most importantly why is31 is chosen as value k? The standard for plants is K=21. Please inclue an explanation of why this value of k was chosen.

 Also, in the first paragraph of the Background section, the grammar in the sentence “Usually found around oligotrophic to mesotrophic acidic waters in can also occur in  nutrient-poor calcareous conditions” is incorrect. Please revise to “Usually found around oligotrophic to mesotrophic acidic waters, it can also occur in  nutrient-poor calcareous conditions”

 In Methods, revise how equipment and protocols are cited. While protocols are cited with an article, equipment and kits should be cited with: company, city, country in brackets. For example in “HMW DNA was sheared into an average fragment size of 12–20 kb in a Megaruptor 3 system ( Bates et al., 2023).”  You should cite the equipment and then say and cite which protocol you are following: “HMW DNA was sheared into an average fragment size of 12–20 kb in a Megaruptor 3 system (company, city, country ) following Bates et al., 2023 protocol. Then please include the citation of SAMtools 

 Finally, as a minor detail indicate what WSI stands for, the first time it is mentioned

Are sufficient details of methods and materials provided to allow replication by others?

Yes

Is the rationale for creating the dataset(s) clearly described?

Yes

Are the datasets clearly presented in a useable and accessible format?

Yes

Are the protocols appropriate and is the work technically sound?

Yes

Reviewer Expertise:

Bioinformatics and Cyperological knowledge

I confirm that I have read this submission and believe that I have an appropriate level of expertise to confirm that it is of an acceptable scientific standard.

# Reviewer report: Reviewer response for version 1

**DOI:** 10.21956/wellcomeopenres.26340.r137674

**Published:** 2025-11-20

**Authors:** Alexander M. C. Bowles

**Affiliations:** 1 University of Oxford, Oxford, England, UK

I read with great interest the manuscript titled “The genome sequence of Bottle Sedge, Carex rostrata Stokes”. This paper presents sequencing and assembly of the *Carex rostrata* genome as part of the Darwin Tree of Life (DToL) project. The data are well presented, whilst the methods and analyses are appropriate and well reported. Overall, this genome is a valuable resource for studying the evolution of sedges.

 A minor comment concerns the BUSCO scores:

 Given the high quality of the genome, 23.8% missing BUSCO genes seems a little high. Is the BUSCO Poales db optimised for all Poales? By my count, eight Carex genomes have been published. These have used the BUSCO Embryophyta/ Viridiplantae dataset to assess genome quality, returning higher completeness scores.

 C. breviculmis: 99% BUSCO Virid https://doi.org/10.1093/aob/mcae085

 C. cristatella 96.1%, C. scoparia 96.4% BUSCO Embryophyta https://doi.org/10.1093/g3journal/jkac211

 C. pumila 91.76% BUSCO Embryophyta https://doi.org/10.3390/genes14112063 

 C. parvula 85.9%, C. kokanica 88.2% BUSCO Embryophyta https://doi.org/10.1038/s41598-022-08783-z 

 C./Kobresia myosuroides 95% BUSCO Embryophyta https://doi.org/10.1093/dnares/dsac049 

 C./Kobresia littledalei 86.2 BUSCO Embryophyta https://doi.org/10.1038/s41597-020-0518-3

Are sufficient details of methods and materials provided to allow replication by others?

Yes

Is the rationale for creating the dataset(s) clearly described?

Yes

Are the datasets clearly presented in a useable and accessible format?

Yes

Are the protocols appropriate and is the work technically sound?

Yes

Reviewer Expertise:

Plant genome sequencing, comparative genomics and evolution

I confirm that I have read this submission and believe that I have an appropriate level of expertise to confirm that it is of an acceptable scientific standard.

# Reviewer report: Reviewer response for version 1

**DOI:** 10.21956/wellcomeopenres.26340.r121175

**Published:** 2025-04-18

**Authors:** María Sanz-Arnal

**Affiliations:** 1 Universidad Pablo de Olavide, Sevilla, Spain

This manuscript presents a chromosomally complete genome assembly for *Carex rostrata* Stokes, a widespread wetland sedge species in boreo-temperate regions. This work is collected under the Darwin Tree of Life project, which aims to sequence all species from the British Isles.

 The authors comprehensively reviewed the nuclear, plastid, and mitochondrial genomes, as well as genome sequencing, assembly, and quality evaluation. The genome was sequenced using PacBio HiFi long reads, scaffolded with Hi-C data, and supported by RNA-Seq. The quality of the genome assembly is excellent, and the dataset is thoroughly documented. This genome represents a valuable resource for studies in genomics and hybridisation within *Carex* and Cyperaceae.

 I considered this study was really well conducted. However, I have a few minor comments that could help make the manuscript clearer.

**MINOR COMMENTS:**

 1. **Taxonomic placement**: to further complete the taxonomic context, it would be beneficial to indicate the section (sect. Hirta Clade, before Vesicariae) to which the *Carex rostrata *belongs (see Global Carex Group, 2021), given the taxonomic complexity of the genus and the utility of this detail for systematic comparisons.

 2 **. BUSCO interpretation**: the manuscript could benefit from a brief contextualisation of the BUSCO scores, for instance, by comparing them to related sedges (Cyperaceae) or Poales genomes. In other words, an explanation of the biological interpretation of these differences (e.g. genome heterozygosity, diploid representation...) would help contextualise the values for readers less familiar with genome assembly evaluation. Acknowledge if missing BUSCO genes might stem from biological or technical factors.

 3. **Figure 1**: within the caption of Figure 1 could appear the ID of the specimen and even the code of the herbarium where it is deposited.

Are sufficient details of methods and materials provided to allow replication by others?

Yes

Is the rationale for creating the dataset(s) clearly described?

Yes

Are the datasets clearly presented in a useable and accessible format?

Yes

Are the protocols appropriate and is the work technically sound?

Yes

Reviewer Expertise:

Taxonomy, macroevolution, bioinformatics

I confirm that I have read this submission and believe that I have an appropriate level of expertise to confirm that it is of an acceptable scientific standard.

## References

[1] : A framework infrageneric classification ofCarex (Cyperaceae) and its organizing principles. *Journal of Systematics and Evolution* .2021;59(4) : 10.1111/jse.12722 726-762 10.1111/jse.12722
